# Effects of Aspirin and Intrauterine Balloon on Endometrial Repair and Reproductive Prognosis in Patients with Severe Intrauterine Adhesion: A Prospective Cohort Study

**DOI:** 10.1155/2017/8526104

**Published:** 2017-01-30

**Authors:** Yuqing Chen, Lixiang Liu, Yuanna Luo, Minghui Chen, Yang Huan, Ruili Fang

**Affiliations:** ^1^Department of Obstetrics and Gynecology, The First Affiliated Hospital of Sun Yat-sen University, Guangzhou, China; ^2^Department of Gynecology Ward II, Shenzhen Luohu People's Hospital, Shenzhen 518000, China

## Abstract

This study aimed to investigate the effects of estrogen in combination with aspirin and intrauterine balloon on the uterine endometrial repair and reproductive prognosis in patients after surgery for severe intrauterine adhesion (sIUA). We prospectively recruited 114 patients with sIUA. Intrauterine device (IUD) was placed and oral estrogen was administered after surgery. Patients were divided into control group and aspirin group. In addition, patients in aspirin group were subdivided into nonballoon group and balloon group. Results showed that, after therapy, the increase in endometrial thickness of aspirin groups was superior to control group (*P* < 0.05). The scores of intrauterine adhesion and menstruation were significantly improved in balloon group as compared to nonballoon group and control group, and significant differences were also observed between nonballoon group and control group (*P* < 0.05). Of 97 patients, 44.3% became pregnant after surgery, the live birth rate was 27.8%, and the miscarriage rate was 37.2%, but there were no significant differences among three groups (*P* > 0.05). Thus, aspirin may promote the uterine endometrial growth and repair after surgery for sIUA, and IUD in combination with intrauterine balloon may reduce the recurrence of intrauterine adhesion, but their effect on the reproductive prognosis is required to be further studied.

## 1. Introduction

Intrauterine adhesion (IUA) is also known as Asherman's syndrome and refers to the complete or partial atresia of the myometrium and/or cervical canal secondary to the damage to the basal layer of the endometrium [1]. Yang et al. found about 93% of IUA was caused by curettage after miscarriage [2]. In China, the abortion rate is as high as 0.29‰ [3], which makes the IUA nonrare. Currently, IUA is often managed by transcervical resection of adhesion (TCRA) via hysteroscopy and postoperative hormone replacement therapy (HRT; estrogen), but the postoperative recurrence rate is as high as 62.5% in patients with IUA [4]. Thus, it is important to promote the endometrial repair after surgery for severe IUA. Our previous study showed microvascular reconstruction is able to promote the endometrial repair after surgery for severe IUA [5]. In a study on the infertility patients who received in vitro fertilization and embryo transfer, results showed additional low dose aspirin could increase the blood flow of uterine artery and increase the pregnancy rate in the presence of HRT [6]. Currently, aspirin has been widely used in the therapy of recurrent miscarriage [7]. However, few studies have been conducted to investigate whether aspirin can improve the local blood circulation to promote the endometrial repair after surgery for severe IUA.

This cohort study was conducted to investigate whether aspirin could promote the endometrial growth and repair, reduce the recurrence of IUA, and improve the menstruation and reproductive prognosis after surgery for severe IUA. Menstruation was evaluated with a method similar to visual analogue scale (VAS) in which the menstruation was assessed by the patients themselves with 0 as amenorrhea and 100 as normal menstruation. This evaluation avoids the vague terms (such as large or small menstrual blood volume) in previous evaluations and considers the individual difference in menstrual blood volume and different understanding about the menstrual blood volume. Thus, this evaluation is easy to master, quantify, and analyze.

## 2. Materials and Methods

### 2.1. Ethics Committee Approval and Patient Consent

This study was approved by the Ethics Committee of the First Affiliated Hospital of Sun Yat-sen University (number 2015-122) and informed consent was obtained before study. This study has been performed in accordance with the principles of Declaration of Helsinki.

### 2.2. Patients

**Figure 1:**
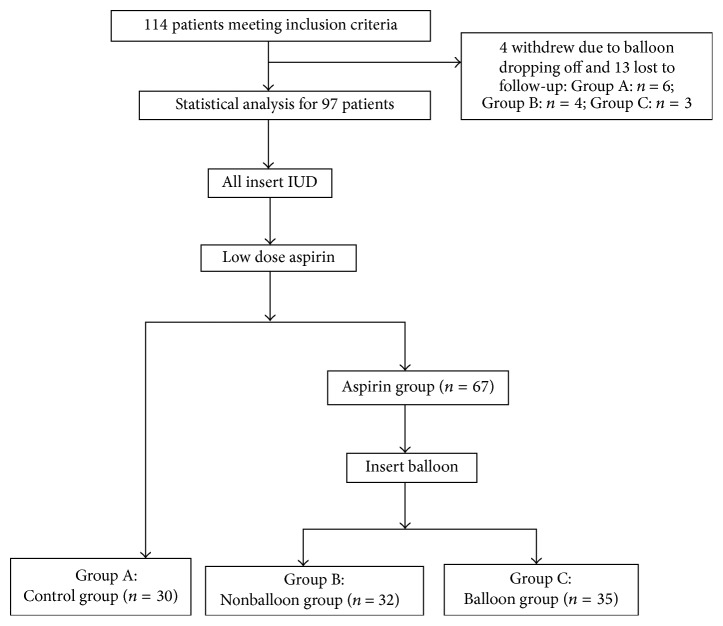
Flowchart of the study design.

A total of 114 patients who received hysteroscopy in the Department of Obstetrics and Gynecology of the First Affiliated Hospital of Sun Yat-sen University and were diagnosed with severe IUA according to the criteria of American Fertility Society (AFS) [8] were recruited into present study, of whom 13 were lost to follow-up and 4 withdrew from this study due to early dropping off or retrieval of balloon. Thus, 97 patients were finally included for analysis (Figure 1). Inclusion criteria were as follows: (1) Preoperative AFS score was ≥9; (2) the prior menstrual cycle was regular, and the sex hormone was normal; (3) patients had fertility requirement; (4) male semen examination appeared normal; (5) there were no severe systemic diseases and no contradictions to aspirin, estrogen, and surgery. Exclusion criteria were as follows: (1) Preoperative AFS score was <9; (2) prior menstrual cycle was irregular and sex hormone was abnormal, or patients had endocrine factors that caused amenorrhea, menstrual reduction, and infertility; (3) patients had no fertility requirement; (4) patients had male factor infertility; (5) patients had contradictions to estrogen and aspirin such as cancers (breast cancer and endometrial cancer), thrombotic diseases, allergy to antipyretic analgesics, severe liver injury, hypoprothrombinemia, vitamin K deficiency, hemophilia, thrombocytopenia, and gastric or duodenal ulcer and asthma. Of 97 patients, 42 received hysterosalpingography before surgery, “irregular uterine morphology, rough uterine edge, and irregular intrauterine filling defect” were reported in 30 patients (71.4%), and “abnormal uterine morphology, double uterus, rudimentary horn, or uterine septum” was reported in 12 patients (28.6%). All the patients received ultrasonography of the uterus. Manifestations of IUA (such as thin endometrium, discontinuity of endometrium, and local intrauterine fluid) were reported in 58 patients (59.8%), inconsistence between endometrium and menstrual cycle was reported in 30 patients (30.9%), uterine malformation, double uterus, rudimentary horn, or IUA was reported in 6 patients (6.1%), and no obvious abnormalities were reported in 3 patients (3.1%).

### 2.3. Methods

TCRA was performed at 3–7 days after menstrual cycle. The time of surgery was not limited in patients with amenorrhea, but it was performed as soon as possible. Surgery was done under general anesthesia by an experienced surgeon. Hysteroscopic device with automatic continuous infusion (STORZ, Germany) was used for surgery. The adherent tissues were bluntly separated and electronically cut to recover the normal morphology of the uterus as complete as possible. After surgery, a round intrauterine device (IUD) was placed. In balloon group, a uterus-shaped balloon was placed in the uterus with the balloon tail being cut at the level of cervix. The balloon was retrieved at 2 weeks after surgery. Second-look hysteroscopy was carried out in the early proliferative phase after 1 to 3 months of surgery to assess the extent and severity of any reformed adhesions. If intrauterine adhesion was not observed, IUD was removed; if film-like adhesion was observed, blunt separation was performed; if severe adhesion was present, patients should be hospitalized and received TCRA again. Hysteroscopy before admission and at 3 months after surgery was performed by the same surgeon who was blind to the grouping in this study.

*Postoperative Medication*. After surgery, estradiol valerate was administered at 3 mg/d thrice daily. At the last 10 days, additional medroxyprogesterone was administered at 10 mg/d for artificial cycle therapy for a total of 3 cycles. In Groups B and C, additional aspirin was administered at 1 mg daily for a total of 3 cycles during the artificial cycle therapy. In Group A, artificial cycle therapy alone was performed after surgery.

### 2.4. Statistical Analysis

Statistical analysis was performed with SPSS version 20.0. Categorical variables were presented as percentages and were compared using the chi-squared test or Fisher's exact test. Continuous variables were presented as *X* ± *S* or median. After the Kolmogorov Smirnov test, continuous variables were compared using one-way ANOVA test or the Kruskal-Wallis test, and changes after therapy were compared within groups using the Wilcoxon matched-pairs signed ranks sum test and between groups using the Mann–Whitney *U* test. A value of *P* < 0.05 was considered statistically significant.

## 3. Results

### 3.1. General Conditions and Clinical Characteristics

The general conditions and clinical characteristics of patients in 3 groups are shown in Table 1. No significant differences were observed among these groups in the above conditions and characteristics (*P* > 0.05). Complications such as massive hemorrhage and water intoxication were not present during surgery. Of these patients, uterine perforation occurred in 3 patients, and then uterine repair was performed by laparoscopy. Pelvic infection was not observed after surgery, and vaginitis was found in 13 patients (Table 1). 57 patients received laparoscopy and hysteroscopy, as well as bilateral tubal hydrotubation; pelvic lesions were found in 41 patients and then managed by surgery; 4 patients with pelvic endometriosis were managed by fulguration; pelvic adhesiolysis was performed in 6 patients by laparoscopy; resection of ovarian teratoma was performed in 4 patients; tubal plastic surgery was done in 3 patients; bilateral tubal ligation was done in 3 patients; resection of tubal mesangial cyst was performed in 5 patients; pelvic adhesiolysis and fulguration of ectopic endometrial lesions were performed in 5 patients; pelvic adhesiolysis and resection of ovarian cyst were done in 3 patients; pelvic adhesiolysis and tubal plastic surgery were done in 3 patients; pelvic adhesiolysis and bilateral tubal ligation were done in 4 patients. Hysteroscopic surgery alone was done in the remaining 40 patients.

### 3.2. Endometrial Thickness Determined before and at 3 Months after Surgery by Ultrasound Examination

The endometrial thickness was detected by ultrasound examination in the ovulation phase before and 3 months after surgery. The endometrial thickness in three groups increased significantly as compared to that before surgery (*P* < 0.05). The difference in endometrial thickness before and after surgery was 0.66 ± 1.76 mm in Group A, 1.76 ± 1.63 mm in Group B, and 1.78 ± 1.77 mm in Group C showing significant difference among them (*P* = 0.011) (Table 2).

In addition, the endometrial thickness in Group B and Group C was thicker than that in Group A (*P* = 0.030 and *P* = 0.022, resp.), but there was no significant difference between Group B and Group C (*P* > 0.05). This suggests that aspirin is helpful for postoperative increase in endometrial thickness, but the effect of balloon increasing endometrial thickness is required to be further confirmed.

### 3.3. Postoperative Reexamination by Hysteroscopy

In three groups, the AFS score at 3 months after surgery reduced significantly when compared with that before surgery (*P* < 0.000). The median difference in AFS score before and after surgery was 6 in Group A (range: 3–10), 7 in Group B (range: 5–10), and 8 in Group C (range: 5–10), showing marked difference among the three groups (*P* < 0.000) (Table 2). The reduction in AFS score in Group C was higher than that in Group B and Group A (*P* = 0.011 and *P* < 0.000, resp.), and there was significant difference in the reduction in AFS score between Group B and Group A (*P* = 0.016).

### 3.4. Menstruation after Surgery

A method similar to VAS was employed for the evaluation of menstruation with 0 as amenorrhea and 100 as normal menstruation. The menstruation score was recorded before and after surgery. The mean menstruation score after surgery increased significantly in three groups as compared to that before surgery (*P* < 0.000). The difference in menstrual score before and after surgery was 22.50 ± 24.52 in Group A, 38.91 ± 28.70 in Group B, and 55.37 ± 25.11 in Group C showing marked difference among the three groups (Table 2). The increase in menstrual score after surgery in Group C was significantly higher than that in Group B and Group A (*P* = 0.035 and *P* < 0.000, resp.), and significant difference was also observed between Group B and Group A (*P* = 0.046).

### 3.5. Pregnancy in the Three Groups

All the patients received contraception before reexamination by hysteroscopy. After second-look hysteroscopy, patients were followed up once every three months by telephone. After surgery, a total of 41 patients became pregnant with the overall pregnancy rate of 44.3% (43/97). Of these patients, the live birth rate was 27.8% (27/97) and miscarriage rate was 37.2% (16/43). The mean time from surgery to pregnancy was 14.2 ± 5.6 months. Natural conception was noted in 28 patients and IVF in 15 patients. Of pregnant women, 1 developed placenta accreta and postpartum hemorrhage and 1 developed placenta previa and postpartum hemorrhage. Of these 41 patients, ultrasonography showed the mean endometrial thickness was 7.10 ± 2.40 mm (range: 5–12 mm). In women with live birth, the mean endometrial thickness was 7.22 ± 2.32 mm. The pregnancy rate and live birth rate were comparable among the three groups (*P* > 0.05).

### 3.6. Postoperative Side Effects

After surgery, breast tenderness was found in 23 patients (Table 1); mild liver dysfunction was noted in 4 patients but resolved after therapy discontinuation; mild contact bleeding of the gingiva was found in 3 patients after medication of aspirin; petechiae of unknown causes in the skin was found in 2 patients, but coagulation function examination and routine blood test appeared normal, and it resolved after therapy discontinuation; mild abdominal pain was noted in 3 patients but was tolerable. Venous thrombosis and severe adverse effects were not observed.

## 4. Discussion

IUA is a rare disease in the Department of Obstetrics and Gynecology. In China, the abortion rate is as high as 0.29‰, which is close to the rate in Estonia which has the highest abortion rate (0.30‰) [3]. This makes IUA nonrare in China. In case of severe IUA, the basal layer of the endometrium is significantly disrupted. The regeneration capability of endometrium and endometrial glands is poor and the endometrial receptivity is poor [9]. Thus, the clinical prognosis of IUA is poor, and the pregnancy rate and live birth rate are relatively low (Table 3). Currently, IUA is managed mainly by postoperative placement of intrauterine balloon [10], IUD [11], intrauterine injection of sodium hyaluronate [12], and amniotic membrane transplantation [13]. Although these treatments may prevent IUA to a certain extent, the pregnancy outcome is still not satisfactory [14]. Thus, it is imperative to improve the postoperative uterine repair, prevent postoperative recurrence of IUA, and improve the reproductive prognosis of patients with IUA.

### 4.1. Role of Aspirin in the Postoperative Endometrial and Morphological Repair in Severe IUA Patients

Currently, postoperative estrogen is recommended for the prevention of postoperative adhesion, which is also found to promote the endometrial repair [15]. However, estrogen treatment is a double-edge sword: it not only promotes the endometrial repair but also induces the fibrous formation and thrombosis. Aspirin is acetyl salicylic acid and can inhibit the platelet aggregation and reduce the activity of prostaglandin (PG) synthetase, exerting antithrombolic effect and antivasospasm effect. Hsieh et al. [16] found that low dose aspirin was able to improve the uterine artery blood flow in the infertility patients with thin endometrium and significantly improve the clinical pregnancy rate. Our previous study showed microvascular reconstruction was able to promote the endometrial repair of severe IUA patients after surgery [5]. Results from present study showed additional aspirin was better to increase the endometrial thickness, reduce AFS score, and improve menstruation (Group B and Group C) as compared to patients without postoperative medication of aspirin (Group A). These findings indicate that aspirin is able to promote the microvascular formation in the endometrium of patients with severe IUA after surgery, improve the local blood circulation, improve the endometrial growth and repair, reduce the recurrence of IUA, and improve the menstruation.

In respect of pregnancy outcome after surgery, the pregnancy rate and live birth rate were comparable among the three groups (*P* > 0.05), but the increase in endometrial thickness is closely related to the clinical pregnancy rate and live birth rate [17, 18]. There is evidence showing that the endometrial thickness should be at least 7 mm for supporting the embryonic implantation [19]. The pregnancy rate in women with endometrial thickness of <7 mm is lower than those with endometrial thickness of >7 mm, and the miscarriage rate in women with endometrial thickness of <7 mm is also relatively high [20]. In our study, the endometrial thickness was 6.95 ± 1.62 mm in Group B and 6.90 ± 1.62 mm in Group C after therapy. Although the endometrial thickness was not thicker than 7 mm, it increased significantly as compared to that before surgery (*P* < 0.001). In the present study, the mean endometrial thickness was 7.10 ± 2.40 mm in 41 patients (range: 5–12 mm). In patients with live birth, the mean endometrial thickness was 7.22 ± 2.32 mm (range: 5–12 mm). This indicates that endometrial thickness and postoperative monitoring of endometrial thickness are helpful for the evaluation of endometrial receptivity and determination of feasibility of pregnancy. In addition, the recurrence and severity of IUA may also affect the postoperative reproductive prognosis [21].

The improvement of uterine morphology is able to improve the endometrial receptivity and create conditions for the embryonic implantation, leading to the improvement of fertility [9]. Thus, aspirin is promising to improve the long term reproductive prognosis in IUA according to its ability to improve the endometrial thickness and prevent IUA.

### 4.2. Effects of Intrauterine Balloon on Postoperative Recurrence of IUA in Patients with Severe IUA

After different hysteroscopic treatments, the time of endometrial repair is also different. After TCRA, the endometrial repair usually requires 1-2 months [2]. Following TCRA, IUD and balloon in the uterus may provide a support for the uterus to prevent IUA and spare time and space for the endometrial repair. Our results showed the reduction in AFS score and improvement of menstruation in Group C (balloon and IUD) was better than in Group B (IUD alone) (*P* < 0.000), indicating that balloon and IUD are better to recover the uterine morphology and volume, reduce the recurrence of IUA, and improve the menstruation. IUD alone is not enough for supporting the uterus because its area is limited, IUA is possible at the center of IUD, and the repaired endometrium might capsule the IUD. Additional balloon is better to separate the central and peripheral uterus, which creates conditions for the endometrial repair. In addition, 2 weeks after surgery when the balloon was retrieved is a key time point for the endometrial repair.

Lin et al. [10] speculated that intrauterine balloon could not increase the intrauterine bacteria within 30 days. Generally, intrauterine balloon is removed at 1 week after surgery [22]. In the present study, the balloon was removed at 2 weeks after surgery considering the risk for intrauterine infection, and subsequently the uterus was supported by the IUD. According to our results, the timely removal of balloon tail after placement and retrieval of balloon at 2 weeks after surgery could not increase the incidence of vaginitis and pelvic inflammation, suggesting that this method is safe. In addition, in severe IUA patients with placement of balloon and amniotic membrane, the pregnancy rate was 23.3% and miscarriage rate was 60% [13]. In our study, the pregnancy rate was 44.3% and miscarriage was 37.2% in patients with placement of balloon and IUD, which were higher than the above reported. This discrepancy might be explained as follows: (1) the balloon stayed in the uterus for a longer time in our study: 3–5 days versus 2 weeks, and thus the balloon had enough time to isolate the uterine wall for the endometrial repair; (2) the balloon was injected with 3–5 ml of water in previous study, which was not done in our study. This might reduce the oppression of water filled balloon on the uterus and avoid the influence of balloon on the blood flow of the uterus; (3) the IUD not only prevents the early dropping off of balloon but also allows the complete expansion of the balloon, which may completely isolate the uterine wall; (4) additional aspirin is able to promote the endometrial repair; the major weakness of our prospective cohort study is its observational design with a small sample size, so the results need to be further confirmed by a larger randomized controlled trial.

### 4.3. Limitations

One possible weakness of this study is that there were cotreatments including operative laparoscopic procedures with possible unequal distribution among the comparison groups. Laparoscopic guidance is reported to aid hysteroscopically directed division of severe IUAs and enable concurrent inspection of the pelvic organs [23–25]. AAGL (Advancing Minimally Invasive Gynecology Worldwide) Practice Report suggests that there is no evidence that hysteroscopic adhesiolysis guided by laparoscopy prevents uterine perforation or improves clinical outcome; however, such an approach used in appropriately selected patients may minimize the consequences if perforation occurs [26]. Therefore, we do not routinely perform laparoscopic treatment at the same time, unless there are indications of operative laparoscopic procedures. However, all groups were similar with respect to the types and frequencies of concomitant factors; therefore, it is reasonable to make comparisons between the groups. In addition, all women were evaluated and managed by the same clinician.

Another possible weakness of this study is the lack of a predefined power calculation. Small studies tend to report larger benefits than larger studies, which is called small-study effects [27, 28], which may overestimate the role of aspirin and intrauterine balloon in the reformation of IUA. Power analysis can be used to calculate the minimum sample size required so that one can be reasonably likely to detect an effect of a given. Therefore, we believe a large sample randomized controlled trial with a predefined power calculation is warranted to further confirm the results of the study.

Third, some women conceive spontaneously as opposed to others treated with IVF. However, whether patients use assisted reproductive technologies is beyond our control. But we would advise patients to use assisted reproductive technologies if necessary.

## 5. Conclusions

Taken together, severe IUA should be managed with comprehensive measures. TCRA is a prerequisite for adhesiolysis: to promote endometrial repair is a key, and prevention of IUA recurrence is key point, all of which are complementary to each other and helpful for the improvement of therapeutic efficacy. In our study, balloon and IUD are placed after TCRA and protect and isolate the injured endometrium, which spare time and space for the endometrial repair. In addition, oral estrogen and aspirin are also beneficial for the endometrial regeneration and repair, which reduce the postoperative recurrence of IUA, improve menstruation, and finally improve the reproductive prognosis.

## Figures and Tables

**Table 1:** Baseline characteristics of the participants.

	Group A (*n* = 30)	Group B (*n* = 32)	Group C (*n* = 35)	*P* value
Age (y)^*∗*^	30.77 ± 4.40	31.44 ± 5.30	30.94 ± 4.87	0.853
Pregnancies^*∗∗*^				0.710
0	2 (6.7%)	5 (15.6%)	6 (17.6%)	
1	9 (30.0%)	8 (25.0%)	5 (14.7%)	
2	10 (33.3%)	10 (31.2%)	12 (35.3%)	
>2	9 (30.0%)	9 (28.1%)	11 (31.4%)	
Miscarriages^*∗∗*^				0.811
0	2 (6.7%)	5 (15.6%)	6 (17.6%)	
1	11 (36.7%)	9 (28.1%)	8 (23.5%)	
2	8 (26.7%)	9 (28.1%)	11 (32.4%)	
>2	9 (30.0%)	9 (28.1%)	9 (26.5%)	
Deliveries^*∗∗*^				0.802
0	27 (90.0%)	28 (87.5%)	29 (85.3%)	
1	3 (10.0%)	3 (9.4%)	4 (11.8%)	
2	0 (0.0%)	1 (3.1%)	1 (2.9%)	
D&C^*∗∗*^				0.635
0	13 (43.3%)	11 (34.4%)	8 (23.5%)	
1	6 (20.0%)	9 (28.1%)	11 (32.4%)	
2	6 (20.0%)	7 (21.9%)	11 (32.4%)	
>2	5 (16.7%)	5 (15.6%)	4 (11.8%)	
Concomitant laparoscopy^*∗∗*^				0.329
Yes	20 (66.7%)	19 (59.4%)	18 (51.4%)	
No	10 (33.3%)	13 (40.6%)	17 (48.6%)	
Tubal patency^*∗∗*^				0.860
Bilateral unobstructed	20 (66.7%)	23 (71.9%)	22 (62.9%)	
Unilateral unobstructed	5 (16.7%)	5 (15.6%)	5 (14.3%)	
Bilateral obstructed	5 (16.7%)	4 (12.5%)	8 (22.9%)	
Postoperative vaginitis^*∗∗*^	4 (13.3%)	3 (9.4%)	6 (8.6%)	0.802
Postoperative breast tenderness^*∗∗*^	6 (20.0%)	8 (25.0%)	9 (25.7%)	0.843

*Note*. D&C: dilation and curettage; *∗*: mean ± SD, one-way ANOVA test; *∗∗*: number (percentage), Chi-squared test/Fisher's test.

**Table 2:** A comparison of endometrium thickness, menstrual score, AFS score, and reproductive outcome among the three groups.

Group	Group A (*n* = 30)	Group B (*n* = 32)	Group C (*n* = 35)	*P* value
Endometrium thickness (mm)				
Preoperation	5.43 ± 1.47	5.18 ± 1.31	5.12 ± 1.16	0.606
Postoperation	6.09 ± 1.49	6.95 ± 1.62	6.90 ± 1.62	0.061
Difference	0.66 ± 1.76	1.76 ± 1.63	1.78 ± 1.77	0.011
AFS score				
Preoperation	10 (9–12)	10 (9–12)	10 (9–12)	0.488
Postoperation	4 (0–6)	3 (0–6)	2 (0–6)	0.000
Difference	6 (3–10)	7 (5–10)	8 (5–10)	0.000
Score				
Preoperation	32.00 ± 23.36	27.97 ± 23.52	21.43 ± 20.49	0.162
Postoperation	54.50 ± 15.33	66.87 ± 15.12	76.80 ± 17.50	0.000
Difference	22.50 ± 24.52	38.91 ± 28.70	55.37 ± 25.11	0.000
No pregnancy	17 (56.7%)	21 (65.6%)	16 (54.3%)	0.617
Pregnancy	13 (43.3%)	11 (34.4%)	19 (45.71%)	
Live birth	8 (26.7%)	7 (21.9%)	12 (34.3%)	0.603
Abortion	5 (16.7%)	4 (12.5%)	7 (20.0%)	
Way of pregnancy				
Natural conception	8 (61.5%)	8 (72.7%)	12 (63.2%)	0.825
Assisted conception	5 (38.5%)	3 (27.3%)	7 (36.8%)	

**Table 3:** Measures taken for prevention of postoperative severe IUA and parameters used for evaluation of severe IUA (literature review).

Study and year	Patient number	Antiadhesion therapy					EM (mm)	RM number	RIUAs number	LB number	P number	M number
		IUD	Balloon	Aspirin	HRT	Other						
Chen 1997	7	Y	N	N	Y	Tents	NR	7	NR	2	3	1
Fernandez 2006	71	N	N	N	Y	N	NR	69	56	21	28	7
Amer 2010	45	N	Y	N	Y	Amnion	NR	35	NR	4	10	6
Myers 2012	12	Y	Y	N	N	N	2–8	12	NR	4	6	2
Chen 2013	36	Y	N	N	Y	N	NR	NR	NR	NR	NR	NR
Tsui 2014	4	N	Y	N	Y	Gel	6–9	NR	NR	2	4	2
Mohamed 2015	40	N	Y	N	N	Amnion	NR	15	NR	NR	NR	NR

N: use; Y: no use; NR: not reported/unknown; HRT: hormone replacement therapy; EM: endometrial thickness; IUA: intrauterine adhesion; IUD: intrauterine device; RM: resumption of menses; RIUAs: recurrence of IUAs; LBN: live birth; P: pregnancy; M: miscarriage.
